# Klippel–Feil Syndrome: A Rare Case Report

**DOI:** 10.1155/crra/7334537

**Published:** 2026-05-21

**Authors:** Mustafa Al Jaafar, Alyaa Nooruldeen Shawqi Aljafr

**Affiliations:** ^1^ Department of Internal Medicine, Ibn Zuhur Hospital, Jisr Diyala, Iraq; ^2^ Department of Rheumatology, Ramadi Teaching Hospital l, Ramadi, Iraq

**Keywords:** conservative management, KFS, rare disease

## Abstract

Klippel–Feil syndrome (KFS) is a rare, complex syndrome characterized by abnormal fusion of cervical vertebrae. This case highlights the effectiveness of conservative management in KFS patients without neurological deficits. We reported the case of an 18‐year‐old Iraqi male presenting a short neck, low back hairline, winged right scapula, and scoliosis after physical examination and deformities of cervical spine fusion, kyphoscoliosis and Sprengel′s deformity after imaging tests. Clinical and radiological diagnosis shows KFS. After conservative treatment—including a corrective collar, cervical traction (5 kg), and targeted physiotherapy for 12 months—significant reduction in neck pain and an increase in cervical range of motion were recognized. This case emphasizes the efficiency of conservative management in KFS patients classified as Samartzis Type II with no neurological disabilities.

## 1. Introduction

Klippel–Feil syndrome (KFS) is a rare congenital condition, defined by Maurice Klippel and Andre Feil in 1912, that is associated with the fusion of two or more cervical vertebrae [1, 2]. Its physical symptoms mainly show a short neck, low back hairline, and restricted mobility of the upper spine [3]. However, this triad is presented in only 50% of patients [4]. KFS is prevalent in one of 40,000 births in the world, with a notable percentage in females.

Genetic heterogeneity exists, since different genetic mutations have been implicated in the disease [5]. Phenotypically, KFS is extremely variable clinically, with involvement of more than one organ system. Approximately 70% of the patients have scoliosis, whereas 20%–30% of the patients have Sprengel′s deformity in the form of congenital elevation and underdevelopment of the scapula that leads to reduced mobility of the shoulder and the characteristic neck lump [6]. There may be other skeletal manifestations in the form of abnormalities of the ribs, spina bifida occulta, and neurological deficits in the form of weakness of the lower limbs and urinary incontinence [5]. Besides skeletal abnormalities, KFS may also have nonskeletal manifestations, including congenital heart defects, mainly ventricular septal defects (VSDs), and renal anomalies like renal hypoplasia, agenesis, or hydronephrosis due to obstruction of the ureters [5]. The extent of multisystem involvement is highly variable between individuals, again showing the heterogeneity of the syndrome.

## 2. Case Presentation

An 18‐year‐old Iraqi male was presented to the Rheumatology Department of Baghdad Teaching Hospital in June 2022 suffering from severe neck pain. Written informed consent was obtained from the patient for the publication of this case report and accompanying images.

The patient was one of the four children born to a consanguineous marriage couple. Antenatal history revealed that no antenatal care was received by the mother, and the child was born by normal vaginal delivery at home. Family history for spinal or neural anomalies was absent.

The parents were informed at birth that their child had a congenital VSD, which may close during infancy or early childhood without treatment. Fortunately, the defect closed spontaneously at 2 years of age. They also had been informed that their child had restricted neck movement and deformities of the cervical and thoracic spine. At 12 years, he started complaining of palpitation and exertional dyspnea, and they consulted a cardiologist; an electrocardiograph and transthoracic echocardiography (ECHO) were performed, diagnosing paroxysmal supraventricular tachycardia (SVT). He was initially treated with antiarrhythmic medication until the condition worsened in 2019, when the patient required multiple admissions to the emergency and cardiac care unit due to frequent and severe dyspnea, with a high rate reaching 300 bpm. In 2020, he underwent catheter ablation with good response.

At birth, the patient was diagnosed with VSD, which closed spontaneously at the age of two. At the age of 12, SVT symptoms emerged, leading to catheter ablation in 2020. In June 2022, the patient presented with a severe neck pain. Conservative treatment was initiated immediately, with follow‐ups occurring every 3 months for 1 year, resulting in significant improvement by June 2023.

**Figure 1:**
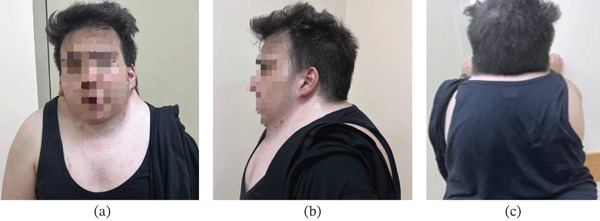
Physical examination: (a) short neck, (b) low posterior hairline, and (c) winged scapula and scoliosis present on right side.

Physical examination demonstrated short neck, low posterior hairline, winged right scapula, and visible scoliotic deformity of the thoracolumbar spine with convexity towards the right side (Figure 1).

The examination of cardiorespiratory and urinary systems revealed no abnormality.

Chest and spine radiographs (x‐rays), computed tomography (CT), and MRI scans were done. Abdominal ultrasounds were prescribed. Electrocardiogram (ECG) and ECHO were also requested.

**Figure 2:**
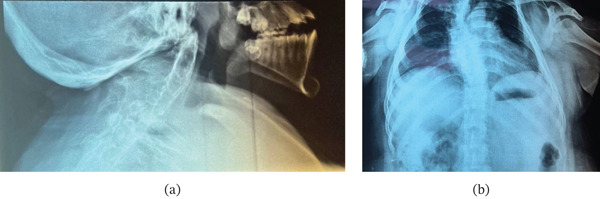
X‐ray: (a) lateral x‐ray showing C2–C5 fusion and (b) AP x‐ray showing thoracolumbar kyphoscoliosis.

X‐ray showed cervical spine fusion, kyphoscoliosis more to the right side with right tracheal displacement with indentation, and with no other abnormality apart from increased cardiac shadow (Figure 2).

**Figure 3:**
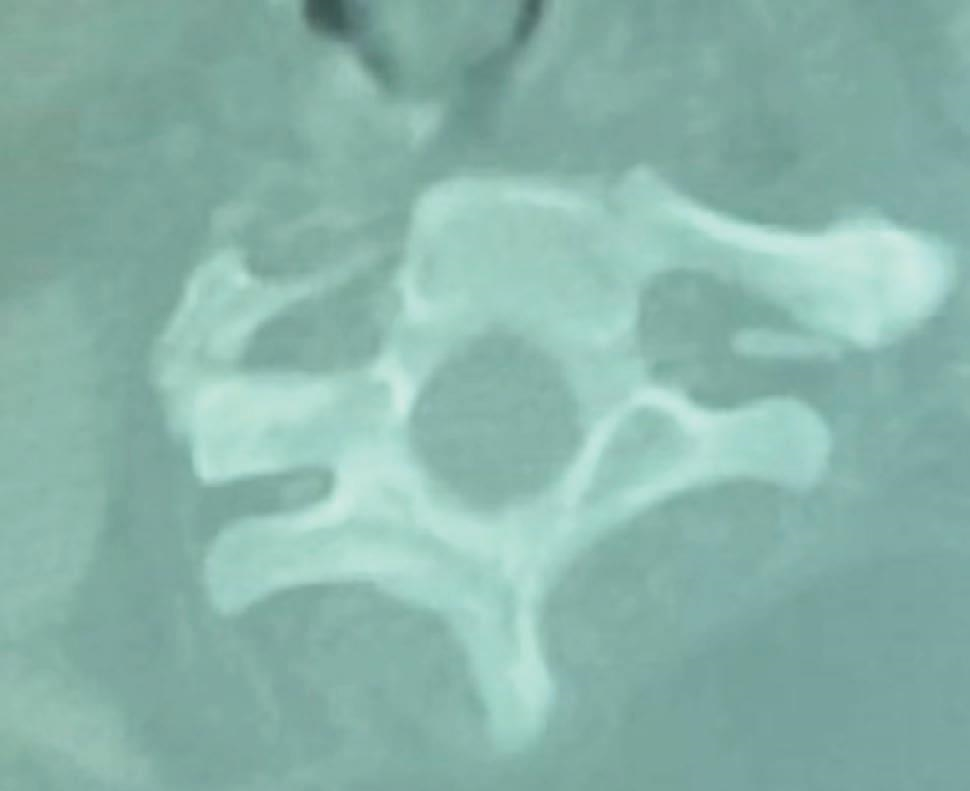
Vertebral wedging.

CT of the spine showed vertebral body fusion involving the upper cervical vertebrae with mild upper dorsal scoliosis with convexity to the right and normal lumbar vertebrae (Figure 3).

X‐ray and CT imaging (Figures 2 and 3) confirmed congenital fusion of the C2 through C5 vertebrae (Samartzis Type II). The fusion at these levels directly correlated with the patient′s restricted neck rotation and chronic myofascial pain.

**Figure 4:**
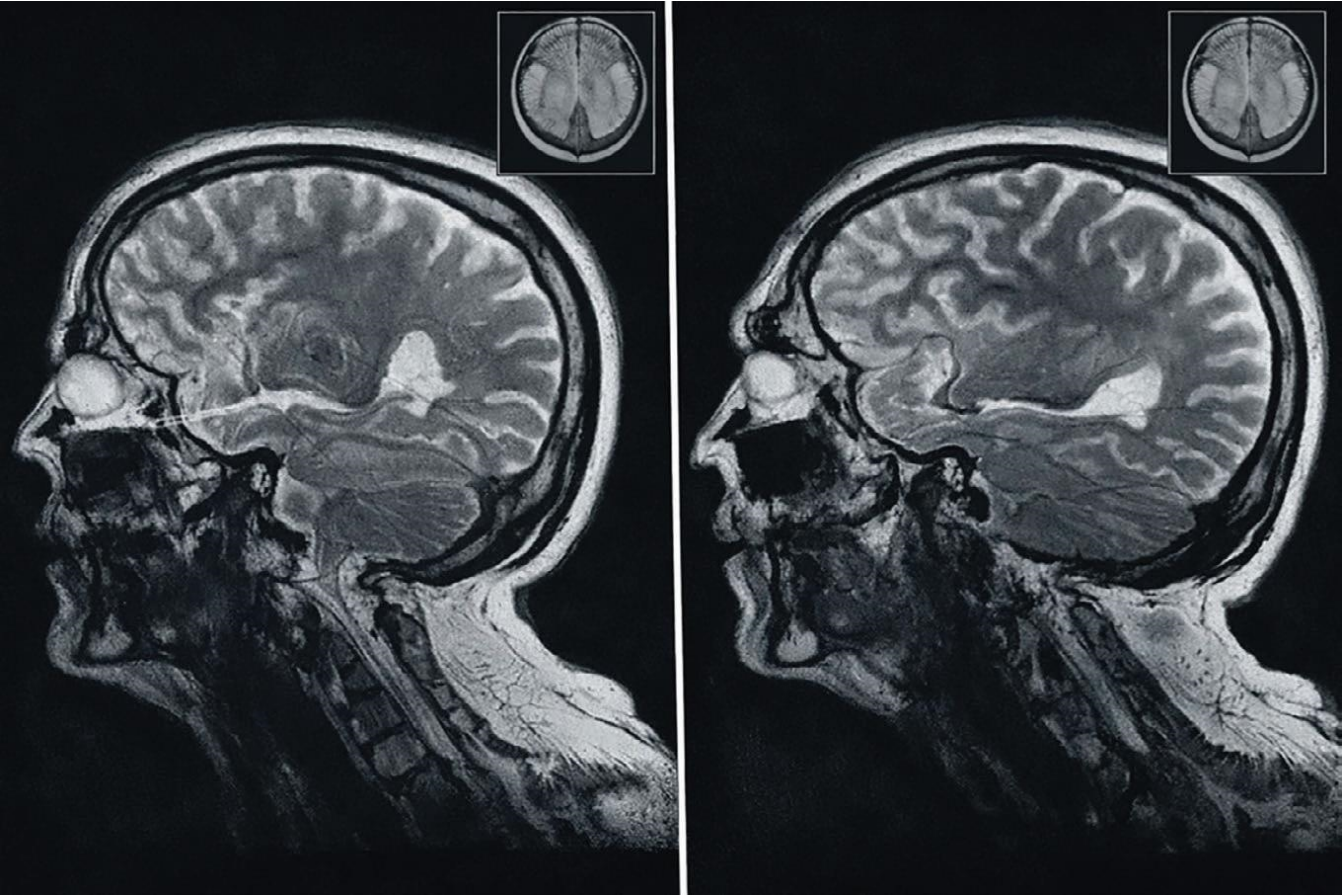
T1‐weighted sagittal MRI of the brain showing mild tonsillar herniation.

MRI of brain revealed mild tonsillar herniation, thickening of left temporal bone, and mild brain atrophic changes (Figure 4).

**Figure 5:**
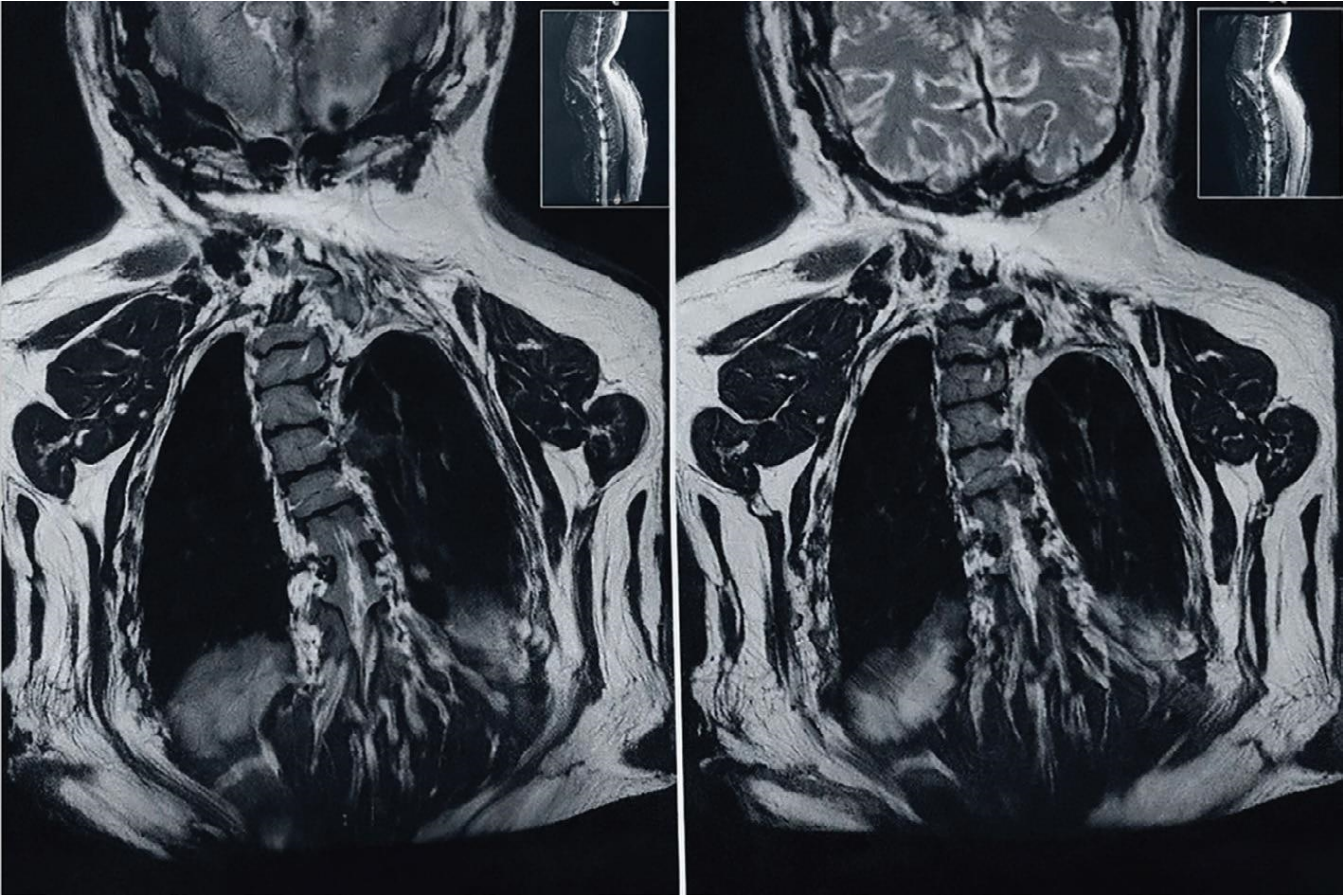
T2‐weighted axial MRI of the cervical spine showing evidence of diastematomyelia at the C3–C4 level.

MRI of the cervical spine revealed diastematomyelia of the upper cervical cord (Figure 5).

ECG and ECHO showed sinus tachycardia and dilated right atrium and ventricle with ejection fraction of 58%.

Abdominal ultrasound was absolutely normal with no internal organ anomaly. No genetic study was performed due to the poor financial condition of the family.

The diagnosis of KFS was reached based on the clinical triad (short neck, low hairline, and restricted range of motion [ROM]) and confirmed via CT/MRI showing vertebral fusion. The presence of Sprengel deformity and a history of VSD and SVT further supported a diagnosis of KFS with multisystem involvement. This confirms the patient as a Samartzis Type II classification based on the single contiguous fusion of C2–C5. Although 3D CT reconstruction is preferred for visualizing complex fusions, standard CT and MRI provide sufficient detail for this diagnosis.

## 3. Treatment and Follow‐Up

**Figure 6:**
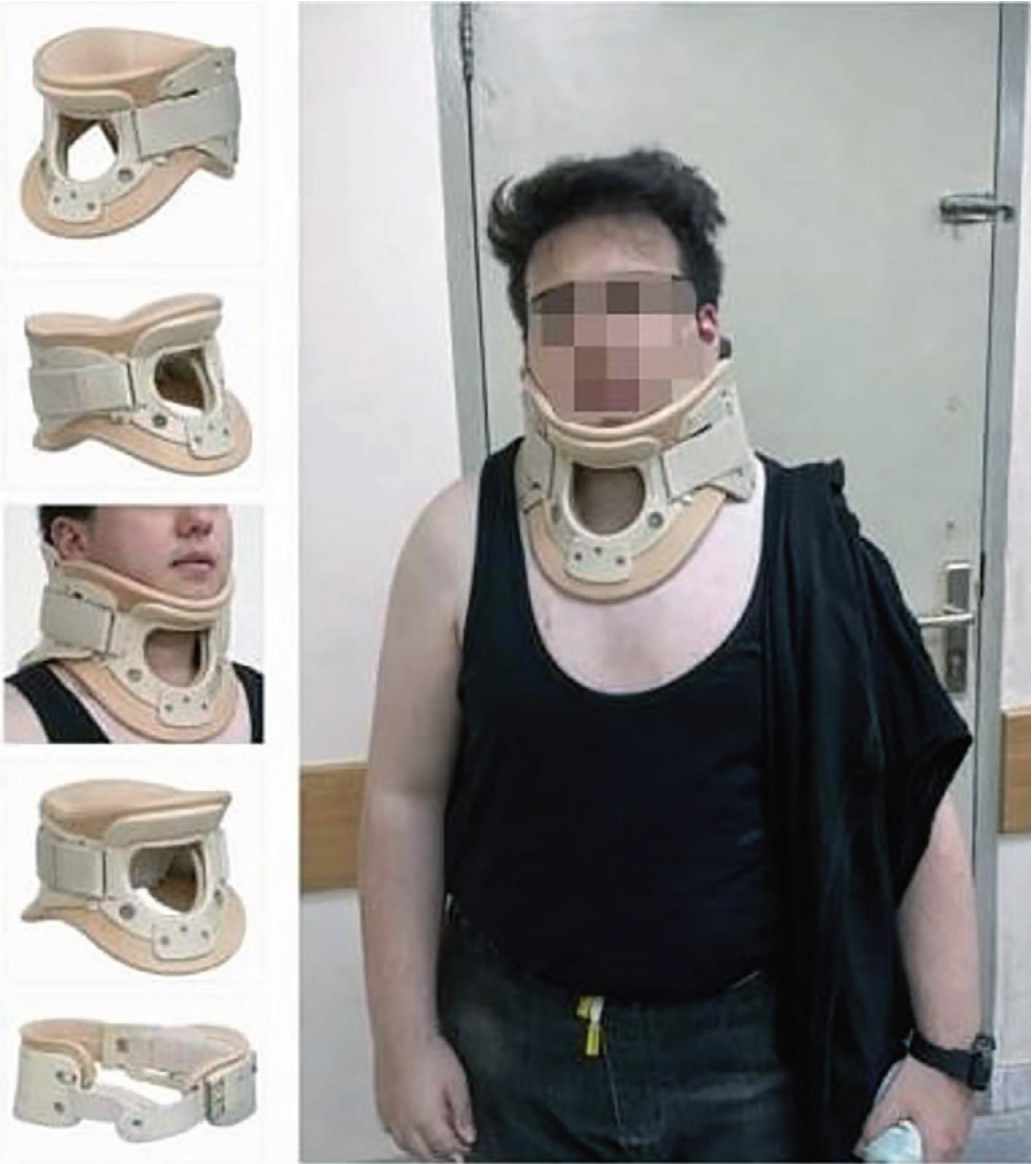
Semirigid orthotic cervical collar (Philadelphia type) used during the conservative treatment protocol, shown standalone and as fitted on the patient.

Regular follow‐up was done to assess his improvement and response to conservative management for a period of 12 months. As he had no neurological deficits or other systemic disease, the primary focus remained on physiotherapy and supportive management. The patient utilized a semirigid corrective cervical collar for 4 h daily to maintain alignment (Figure 6). Manual cervical traction was applied at 5 kg for 20 min daily, performed at home under parental supervision. Physiotherapy focused on isometric neck strengthening and scapular stabilization exercises (3 sets of 10 reps, 5 times daily) to improve mobility and prevent stiffness.

On subsequent follow‐ups, the patient improved gradually in neck mobility and right shoulder function. There was no worsening of symptoms, and he was neurologically intact. Pain and discomfort due to cervical fusion and Sprengel′s deformity were well controlled with the given physiotherapy protocol. The prognosis was reviewed periodically, and the need for long‐term adherence to physiotherapy was stressed.

At latest follow‐up, the patient experienced a significant relief of symptoms and improved functional outcome with no new or worsening symptoms. He continues to have periodic examinations to ensure long‐term stability and follow‐up for potential late‐onset complications of KFS.

No adverse or unanticipated events were reported during the 12‐month course of physiotherapy and cervical traction.

## 4. Discussion

KFS is a rare congenital condition involving cervical vertebrae fusion, commonly characterized by a classic triad of short neck, low posterior hairline, and restricted movements of the neck [7, 8]. This triad is observed in only about 40%–50% of patients; hence, making diagnosis challenging, particularly in asymptomatic patients [3, 9].

The differential diagnosis for this patient included Wildervanck syndrome (due to the cervical fusion) and Turner syndrome (due to the short neck). However, the absence of sensorineural deafness and the presence of the specific C2–C5 fusion confirmed by imaging favored KFS. Unlike cervical spondylosis, the fusion in this case was clearly congenital, lacking degenerative osteophytes at the fused levels.

KFS most frequently occurs together with other congenital abnormalities like Sprengel deformity, scoliosis, genitourinary malformations, congenital heart disease, and neurological anomalies. Due to the heterogeneity of presentation, prompt diagnosis along with a multidisciplinary management approach is very crucial for optimization of patient prognosis.

In our patient, there was a massive cervical fusion and Sprengel deformity but no neurological deficits or systemic involvement. The patient was managed conservatively, with stress laid on physiotherapy, manual cervical traction, and the wearing of a corrective cervical collar. During follow‐up, the patient showed marked relief of symptoms, with enhanced mobility of the neck and function of the shoulder, indicating that nonsurgical management can be a reasonable first‐line treatment in specific cases.

Radiological evaluation is the gold standard for KFS classification [4]. Type II (fusion at a single contiguous segment) typically carries a lower risk of neurological compromise compared with Type III. However, CT/MRI is vital to screen for “hidden” anomalies. For instance, airway management in KFS patients is a known radiological concern; the restricted mobility and potential for atlantoaxial instability and basilar invagination (though not present in this patient) require careful assessment before any anesthetic intervention. Furthermore, sagittal MRI is essential to rule out syringomyelia, which can occur in up to 25% of cases with craniovertebral junction abnormalities. Imaging studies such as x‐rays and MRIs still have a significant role to play in diagnosis and recognition of related anomalies such as spinal cord compression or atlantoaxial instability. However, research indicates that KFS patients have scoliosis with Sprengel deformity, which significantly impacts their quality of life [10–12]. Our patient is a characteristic of this group because the patient had a condition of Sprengel deformity without any general neurological impairment.

The literature indicates that the management of KFS ranges from conservative to surgical, based on the severity of the symptoms [13, 14]. In cases of severe instability or neurological sequelae, surgical options such as spinal fusion can be required; however, these are not risk‐free, with potential nerve damage, arterial injury, and postoperative morbidity. Conservative management, encompassing physiotherapy and cervical immobilization, proved adequately effective in alleviating mild symptoms associated with slight spinal instability [14]. The patient also served as evidence for the efficacy of nonoperative interventions, indicating that supervised physiotherapy along with vigilant monitoring can improve the functional outcomes of patients with KFS.

Due to KFS and its clinical manifestations, treatment needs to be multidisciplinary. Patients need to undergo detailed systemic assessments, including neurological, cardiological, and renal assessments, to ascertain whether there are any underlying comorbid illnesses. The case presented here underlines the importance of treating each patient separately according to his or her special needs and severity of the illness.

Participation of caregivers and patient education are also significant in facilitating compliance with exercises as prescribed and follow‐up treatment over the long term. Conservative treatment was well tolerated by the patient, but long‐term follow‐up is required since KFS may progress to degenerative changes and secondary complications.

A primary strength of this case report is the 12‐month follow‐up period, which allowed for a clear assessment of the efficacy of conservative management. However, a significant limitation was the inability to perform 3D CT reconstruction or genetic testing due to the family′s financial constraints. Although these are not strictly necessary for a clinical diagnosis of KFS, they would have provided more detailed anatomical and hereditary insights.

The patient reported significant satisfaction with the noninvasive nature of the treatment. He noted that the combination of the cervical collar and the specific home exercises allowed him to return to daily activities with a marked reduction in pain and improved neck mobility without the need for surgical intervention.

## 5. Conclusion

The case contributes to the literature by showing how conservative management in the KFS patient with Sprengel deformity can be efficacious without neurologic compromise. KFS is a debilitating disease that requires personalized management. However, our experience shows that some patients may benefit symptomatically and functionally from nonoperative management. There is a need for further research towards the establishment of standardized treatment guidelines and the investigation into genetic factors that may account for disease variability.

## Funding

No funding was received for this manuscript.

## Conflicts of Interest

The authors declare no conflicts of interest.

## Data Availability

The data that support the findings of this study are available from the corresponding author upon reasonable request.
